# The complete chloroplast genome of *Keteleeria davidiana* var. *calcarea* (Pinaceae), an endangered species endemic to China

**DOI:** 10.1080/23802359.2021.1882906

**Published:** 2021-03-01

**Authors:** Jingjian Li, Niya Cheng, Yancai Shi

**Affiliations:** a College of Pharmacy, Guilin Medical University, Guilin, China; b The Chinese Academy of Sciences, Guangxi Institute of Botany, Guilin, China

**Keywords:** *Keteleeria davidiana* var. *calcarea*, chloroplast genome, phylogenetic analysis

## Abstract

*Keteleeria davidiana* var. *calcarea* is an endangered tree with considerable economic potential that used as timber wood for furniture and house construction. However, the natural population of *K. davidiana* var. *calcarea* is very fragmented, which is the cause for its low genetic diversity. In this study, we report the complete chloroplast genome of *K. davidiana* var. *calcarea* using Illumina sequencing. The chloroplast genome size is 117,670 bp in length, harboring a pair of very short inverted repeats (IRs) of 262 bp separated by a large single copy (LSC) sequence of 64,634 bp and a small single copy (SSC) sequence of 53,078 bp. The chloroplast genome *K. davidiana* var. *calcarea* contains 113 genes (74 protein genes, 35 tRNA genes, and 4 rRNA genes) and the overall GC content is 38.6%. The maximum likelihood phylogenetic analysis shows that *K. davidiana* var. *calcarea* is clustered with *K. davidiana* in genus *Keteleeria*. This complete chloroplast genome will help us to understand the evolution of *K. davidiana* var. *calcarea* and lays the foundations for future studies in this species conservation.

*Keteleeria davidiana* var. *calcarea* is a rare tree species which grows mainly in southwestern China. This tree exhibits strong and heavy wood material characteristic, which make it suitable for house construction, furniture manufacture and ship-building (Jiang et al. 2008). At present, due to its vulnerable reproductive capacity couple with excessive logging by human, *K. davidiana* var. *calcarea* has become an endangered species and has been listed as important protected species in China (Xie et al. 2017). In order to protect this species effectively, researchers have paid a lot attention to formulate strategies for the cultivation and conservation of *K. davidiana* var. *calcarea* (Xie et al. 2017). However, there are still few studies regarding to the evolution and phylogeny of *K. davidiana* var. *calcarea* from the molecular level, which we believe to be an important yet underappreciated direction for this species conservation. A complete chloroplast genome sequence of *K. davidiana* var. *calcarea* will contribute to the progress of these works.

Chloroplast genome is often utilized for phylogenetic analysis and domestication studies of higher plants (Nie et al. 2020). The whole chloroplast genome sequences have also been demonstrated the potential to understand structure and functional evolution (Sabater 2018; Cheon et al., 2019; Zha et al. 2020). In the genus *Keteleeria*, the chloroplast genome of some species such as *K. davidiana* and *K. evelyniana* has been reported (Wu et al. 2009; Li et al. 2019), but the chloroplast genome of *K. davidiana* var. *calcarea* has not been reported. Here, we sequenced and analyzed the complete chloroplast genome sequence of *K. davidiana* var. *calcarea* based on the Illumina sequencing data. This study aimed to characterize the complete chloroplast genome sequence of *K. davidiana* var. *calcarea* as a resource for future genetic studies.

Fresh young leaves from one individual of *K. davidiana* var. *calcarea* were collected from Guangxi Institute of Botany, The Chinese Academy of Sciences, Guilin, China (latitude: 25.0677; longitude:110.3037). The total DNA was extracted using the DNeasy Plant Mini Kit (QIAGEN, German). Voucher specimen of *K. davidiana* var. *calcarea* was deposited at the herbarium of Guangxi Institute of Botany (contact person: Yancai Shi, email: shiyancainan@163.com) under the voucher number IBK-SYC-202006). The whole-genomic DNA data was sequenced using the Illumina HiSeq2000 platform, which was then assembled using the program GetOrganelle (Jin et al. 2020) with *K. davidiana* (NC_011930) as reference. The assembled chloroplast genome was annotated by the combination of PGA (Qu et al. 2019) and GeSeq (Tillich et al. 2017). For necessary genes, we manually corrected their positions of start and stop codons and boundaries between exons and introns.

The complete chloroplast genome of *K. davidiana* var. *calcarea* is 117,670 bp in length with a typical quadripartite structure contains a pair of short inverted repeat regions (IRa and IRb) consisting of 262 bp each, a large single copy (LSC) region of 64,634 bp and a small single copy (SSC) sequence of 53,078 bp. The chloroplast genome contains a total of 113 genes, including 74 protein-coding genes, 35 tRNA genes and 4 rRNA genes. The gene content and gene order are similar to the chloroplast genomes of other species in Pinaceae (Yi et al. 2016). Fifty-seven protein coding and 15 tRNA genes are located in the LSC region, while 17 protein-coding, 18 tRNA genes and 4 rRNA are located in the SSC region, respectively. Only one tRNA gene (trnI-CAU) is duplicated and located on the IR regions. All ndh genes have been lost in the genome of *K. davidiana* var. *calcarea* like other chloroplast genomes of family Pinaceae. The overall GC content of *K. davidiana* var. *calcarea* chloroplast genome is 38.6%, which is similar with those of other member of family Pinaceae and general angiosperms (Qian et al. 2013; Yi et al. 2016).

To confirm the phylogenetic position of *K. davidiana* var. *calcarea*, a phylogenomic analysis was performed based on 19 published species within Pinaceae and one out group (*Ephedra foeminea*, Ephedraceae). A total of seventy-two protein coding genes shared by all the species were extracted, and were aligned by using MUSCLE (Edgar 2004). Tree topologies for Maximum likelihood (ML) analysis using RAxML 8.0 software (Stamatakis 2014) based on the GTR + R + I model were congruent with each other and all clades were strongly supported in those trees (–lnL of −192708.384031). The ML tree supported that *K. davidiana* var. *calcarea* and *K. davidiana* cluster as sister in genus *Keteleeria* (Figure 1), which is consistent with the previous studies in Pinaceae (Li et al. 2019; Yi et al. 2016). In conclusion, this published *K. davidiana* var. *calcarea* chloroplast genome will provide a solid foundation for phylogenetic and evolutionary studies in *Keteleeria* and is expected to help us formulate protection strategy for this species conservation.

**Figure 1.**
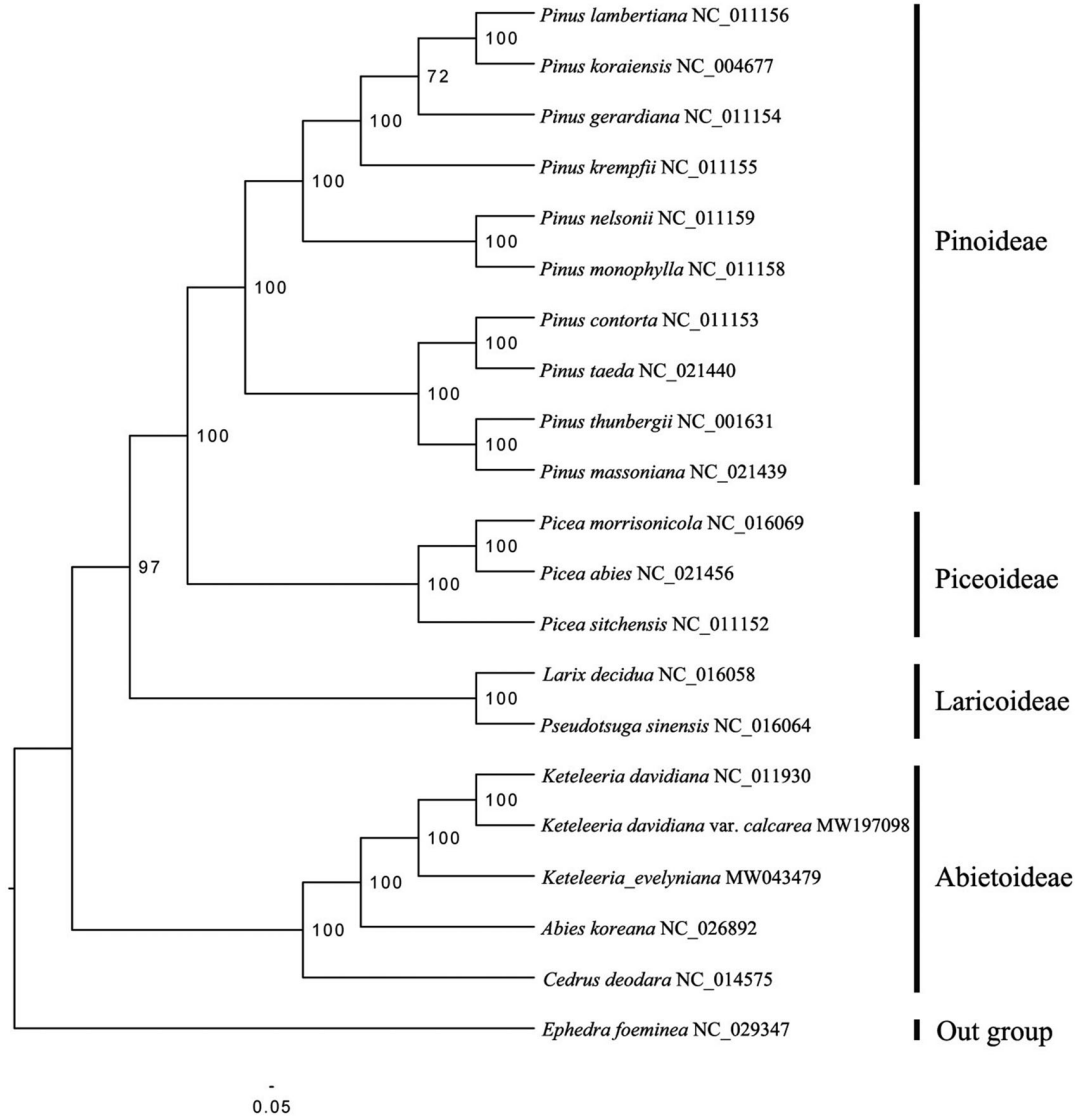
Maximum likelihood phylogenomic tree inferred from *K. davidiana* var. *calcarea* and other 19 species within Pinaceae and one out group species (*E. foeminea*) using complete chloroplast genomes. Bootstrap supports based on 1000 replicates are given at the node.

## Data Availability

The genome sequence data that support the findings of this study are openly available in GenBank of NCBI at (https://www.ncbi.nlm.nih.gov/) under the accession no. MW197098. The associated BioProject, SRA, and Bio-Sample numbers are PRJNA674712, SUB8472759, and SAMN16672654, respectively. Tree file of 21 species and genes for phylogenetic analysis were deposited at Figshare: https://doi.org/10.6084/m9.figshare.13207604.
